# Factors associated with sterilization use among women leaving a U.S. jail: a mixed methods study

**DOI:** 10.1186/1471-2458-14-773

**Published:** 2014-07-31

**Authors:** Megha Ramaswamy, Patricia J Kelly

**Affiliations:** Department of Preventive Medicine and Public Health, University of Kansas School of Medicine, 3901 Rainbow Boulevard, Kansas City, Kansas 66160 USA; School of Nursing and Health Studies, University of Missouri-Kansas City, 2464 Charlotte Street, Kansas City, Missouri 64108 USA

## Abstract

**Background:**

Despite the high rates of reported sterilization use among women who have spent time in correctional facilities, little is known about the context in which women in this population choose this option. The objective of our study was to use both quantitative and qualitative methods to understand factors associated with sterilization use among women leaving a U.S. jail.

**Methods:**

We administered a cross-sectional survey with 102 jailed women who were participating in a study about contraceptive use after release from jail, and then conducted semi-structured interviews with 29 of those women after their release from jail. We used logistic regression and analytic induction to assess factors associated with self-reported sterilization use.

**Results:**

In our cross-sectional survey, one-third of our sample reported a history of sterilization use. Controlling for age and past pregnancies, the only factor associated with sterilization use was physical abuse history before age 16. In semi-structured interviews, we found that women’s primary motivation for sterilization was the desire to limit childbearing permanently, in some cases where other contraceptive methods had failed them. The decision for sterilization was generally supported by family, partners, and providers. Many women who opted for sterilization expressed financial concern about supporting children and/or reported family histories of sterilization.

**Conclusions:**

The decision to use the permanent method of sterilization as a contraceptive method is a complex one. Results from this study suggest that while explicit coercion may not be a factor in women’s choice for sterilization, interpersonal relationship histories, negative experiences with contraceptives, and structural constraints, such as financial concerns and ongoing criminal justice involvement, seem to influence sterilization use among the vulnerable group of women with criminal justice histories. Public health programs that connect women to reproductive health services should acknowledge constraints on contraceptive decision-making in vulnerable populations.

## Background

Women under correctional supervision represent some of the most vulnerable members of society, with up to 45% reporting histories of mental health problems, 40% child physical abuse, 60% forced sexual activity, and 67% reporting partner violence [1–3]. Less than half, but even as low as one-quarter, of women were employed prior to their incarcerations [3–5]. One-third of women in jails and prisons report having completed high school [6].Women who bear the greatest burden of incarceration in the United States, in particular, are disproportionately from racial and ethnic minority groups [1]. Black women in the U.S. are incarcerated at three times the rate of White women, and Latina women are incarcerated at 1.6 times the rate of White women [7].

Compounding these social vulnerabilities, women in the justice system report rates of unintended pregnancy of up to 80%, as a result of erratic contraceptive (66.5%) and condom use (80.4%) [8]. Sixty pecent of women in one study reported not using any contraception while having sex with opposite sex partners in ongoing relationships prior to incarceration, though 72% reported a regular sex partner [3]. These patterns of contraceptive use may be the result of poor access to effective contraceptive services and/or disempowerment in interpersonal relationships. However, it is not lack of desire to limit pregnancies that results in this contraceptive pattern, since 40% of one sample of jailed women reported sterilization as their method of choice for pregnancy prevention [9]. This high rate of sterilization use in a vulnerable population raises the question of whether explicit or implicit coercion might play a role in their contraceptive decision-making.

In the U.S., the abuse of sterilization as a form of eugenics among vulnerable populations, such as young African-American women in the South, Native American women, and developmentally disabled women, has been well established [10–12]. The documentation of these abuses by advocacy groups through general publicity, public hearings, and court cases led to the adoption of the 1979 federal regulations for sterilization [13]. These regulations limit sterilization of women under age 21 and those who are developmentally disabled. A 30-day waiting period after signing of consent was also implemented, as well as restrictions on obtaining consent during childbirth. However, abuse continues, as seen in the recent coercive sterilization of 148 women in a California state prison who were not properly consented about the procedure [14]. Examples of abuse of sterilization are not particular to the U.S., either, as cases have been documented all over the world – in Canada, India, and China, for example [15–17].

Given this history of exploitation of vulnerable groups as it relates to sterilization, in particular among incarcerated women who bear the burden of multiple vulnerabilities – health, social, and economic, we sought to conduct an initial exploratory study of factors associated with sterilization use among women with criminal justice involvement. Incarcerated women have reported high rates of sterilization relative to the general population [9, 18], and in at least one documentd case, a group of incarcerated women were explicitly coerced into undergoing the procedure [14]. Thus, the objective of our study was to use a mixed methods approach to understand factors associated with sterilization use among women leaving a U.S. jail. Our goal was to assess whether explicit or implicit constraints may be associated with this vulnerable group of women’s contraceptive decision-making.

## Methods

We used a mixed methods approach to understanding factors associated with sterilization use among women leaving jail. We conducted a secondary data analysis of a cross-sectional study of women’s contraceptive use after release from jail. After initial analysis of the cross-sectional study data revealed that one-third of the sample had undergone tubal ligations, we recruited a convenience sample of women who participated in the cross-sectional study to complete semi-structured interviews in order to better understand women’s thoughts, feelings, and experiences related to sterilization use. We felt that further exploration of their experiences with sterilization use, even with sampling limitations, was warranted given the prevalence of reported sterilization use in this vulnerable group.

### Study design and sample

#### Jail-based cross-sectional study

We conducted a cross-sectional study in spring and summer of 2011 with a convenience sample of 102 women in an urban county jail in Kansas City, Missouri. These interviews were part of an ongoing longitudinal study of women in these jails and their use of and access to sexual health care after release from jail. Sample size for the cross-sectional study was determined by the parent study’s aim of following a sample of women into the community in order to generate preliminary data about use of and access to sexual and reproductive health care. Women were eligible to participate if they were within one week of release from jail. Recruitment occurred by posting flyers in the women’s housing unit at the jail to publicize the study. We approached each woman who was scheduled for release that day to inquire about study participation and answer questions about the study. Interested women signed a consent form and completed the survey. We did not collect data about the women who chose not to participate in the study, however our sample was similar to all women incarcerated in the jail along race, ethnic, age, and criminal justice characteristics. Women received $10 for their participation in the jail-based survey.

#### Community-based semi-structured interviews with a subsample of participants

In order to further understand women’s motivations and/or reasoning for a tubal ligation, we conducted community-based, post-release, semi-structured interviews with a non-purposive convenience sample of previously enrolled study participants about their experience surrounding sterilization (N = 29). Previously enrolled study participants were chosen for convenience and interviewed in the order they were contacted. Interviews stopped when thematic saturation was reached [19]. Interviews were conducted by a trained master’s student (the same person who conducted most jail-based surveys) at a centrally located city health department and women received $50 compensation for the community-based interview.

Both the cross-sectional study and semi-structured interviews were approved by the Institutional Review Board at the University of Kansas Medical Center.

### Data collection

The 166-item cross-sectional survey included items about sociodemographics, pregnancy, contraceptive history, and incarceration history. The dependent variable, sterilization history, was measured with the question, “Have you ever had a tubal ligation?” Information on health risks common among women in the criminal justice system was collected, including exchange of sex for money, depression, drug dependence, or alcohol problems, and abuse history. Depression was assessed with the CES-D 10 [20]. Drug dependence was assessed using DSMIV criteria. For example, participants were asked six questions about drug use in the year before incarceration that included questions such as: “Did you need to use more drugs to get the same high as when you first started using?” If participants answered “yes” to three out of six criteria, they were classified as “drugdependent^”^ [21]. Alcohol problems were assessed using the CAGE questionnaire. Participants were asked four questions about lifetime alcohol use, such as: “Have you ever felt the need to cut down on drinking?” If participants answered “yes” to two or more questions, they were coded as “at risk for alcohol problems” [22]. We assessed past year intimate partner violence by asking participants if a sex partner had physically hurt, insulted, or screamed at the participant on a regular basis or fairly often in the year before incarceration (adapted from Verbal HITS Scale) [23]. Childhood physical abuse history was assessed by asking participants if they had been hit, pushed or shoved, or kicked or punched before age 16 (Childhood Experiences of Violence Questionnaire) [24]. With questions from the same instrument, we assessed childhood sexual abuse history by asking participants if anyone had done the following things when you didn’t want them to: touch the private parts of your body, make you touch their private parts, threaten or try to have sex with you, or sexually force themselves on you”^19^.

Semi-structured interviews focused on participants’ direct and/or indirect experience with sterilization began with the question: “Have you had a tubal ligation?” For women responding affirmatively, details were asked about age at time of tubal ligation, site of procedure, reasoning for tubal ligation versus alternate forms of birth control, types of people (if any) helping to make the decision (e.g., sex partners, relative, friend), and the level of support of the medical provider leading up to the procedure. Women reporting not having had a tubal ligation were asked whether or not they had ever been interested in having the procedure performed. Those expressing past, present or future interest in a tubal ligation as a form of birth control were asked to detail what types of issues or barriers they faced (e.g., cost, age, interpersonal relationships, irreversibility of procedure, desire for children). For women with no personal experience or interest in a tubal ligation, we asked if the interviewee knew of any other women in their lives with a tubal ligation; they were then asked to provide a second-hand account, including as many details possible, related to another woman’s experience completing a tubal ligation (e.g., age, location, reasoning, support).

### Data analysis

Cross-sectional survey data were analyzed in SPSS and descriptive statistics were generated for all variables. Variables were chosen based on factors associated with sterilization in the general population [18, 25], as well as factors that might be unique or more common in our sample, e.g. drug use history, mental health problems, exchange of sex for money, and criminal justice history [2, 8]. We ran logistic regression models [26] to assess the association of each potential independent variable with the dependent variable of interest – sterilization history. Models for the variables that had significant independent associations with sterilization history (at the p ≤ 0.05 level) were then run, controlling for age and number of pregnancies carried to term, the two factors that were associated with sterilization history in the general population [18, 25] and that were independently associated with sterilization history in our sample.

Qualitative interviews were transcribed verbatim and the transcripts checked against the original audio recordings. Semi-structured interviews were conducted to explore two alternative hypotheses – one, that incarcerated women were explicitly coerced into sterilizations, or two, that incarcerated women opted for sterilization in the face of more implicit constraints. Analytic induction was employed for the analysis, allowing the data to be fit into either of the two hypotheses [27]. Transcripts were reviewed and significant statements and key phrases assigned codes independently by the two authors. Areas of disagreement were discussed and resolved [28]. Coded statements were organized into concept clusters of related content [29]. We then assessed the extent and context in which patterns occurred in the data, when and how observations deviated from our hypotheses, and what significance patterns and deviations were associated with sterilization history [30]. The description of this study conforms to RATS (Relevance, Appropriateness, Transparency, and Soundness) guidelines for qualitative research [31].

## Results

### Participant characteristics

The 102 participants in the cross-sectional survey were on average 34 years old (range 18–60). Seventy-two percent (N = 73) of the women were Black and 16% (N = 16) White. As shown in Table 1, the majority of participants (71%) had a high school education. Only one-third (N = 35) was employed prior to incarceration. The women had spent on average 11 months in jail or prison (range one month to 16 years). Participants reported an average of two pregnancies in their lives (range 0–8). One-third (N = 32) reported having had a tubal ligation. Two-thirds of the women (N = 62) reported having an unintended pregnancy. Twenty-five percent (N = 25) said they had exchanged sex for money, drugs, or life necessities at some point in their lives. Sixty-three percent (N = 64) of the women had current depression according to the CES-D; 36% (N = 37) experienced drug dependence in the past year, according to DSM-IV criteria, and 30% (N = 30) indicated lifetime alcohol problems, as measured by the CAGE questionnaire. Forty-two percent (N = 42) of the women reported past year intimate partner violence. One-third of the women reported child physical abuse (N = 31) and sexual abuse histories (N = 33).

**Table 1:** **Participant characteristics and factors associated with sterilization history, N = 102**

	N (%) or mean (min, max)	Odds ratio (95% CI) for association with sterilization history	Adjusted odds ratio (95% CI) for association with sterilization history*
Age	33.72 (18, 60)	**1.12 (1.06, 1.18)**	–
White	16 (15.7)	1.02 (0.40, 2.59)**	–
Black	73 (71.6)		
American Indian/Alaska	2 (2.0)		
Native	3 (2.9)		
Bi-Racial	1 (1.0)		
Other	7 (6.9)		
Latina			
High school education or more	72 (70.6)	0.58 (0.24, 1.41)	–
Employed prior to incarceration	35 (34.7)	1.00 (0.98, 1.01)	–
Number of pregnancies ever carried to full term	2.10 (0, 8)	**2.10 (1.50, 2.96)**	–
Ever had a tubal ligation	32 (31.4)	–	–
Ever had an unplanned pregnancy	62 (60.8)	1.35 (0.57, 3.23)	–
Ever exchanged sex for money, drugs, life necessities	25 (24.8)	**2.77 (1.08, 7.10)**	1.15 (0.36, 3.71)
Number of months incarcerated in lifetime	10.92 (0, 192)	**1.02 (1.00, 1.04)**	1.01 (0.99, 1.03)
Current depression	64 (62.7)	1.20 (0.50, 2.87)	–
Past year drug dependence	37 (36.3)	**2.33 (0.99, 5.52)**	1.07 (0.36, 3.19)
Lifetime alcohol problems	30 (30.0)	**2.95 (1.19, 7.33)**	1.77 (0.59, 5.29)
Past year intimate partner violence	42 (41.6)	1.37 (0.59, 3.20)	–
Physical abuse history before age 16	31 (30.4)	**2.98 (1.22, 7.26)**	**3.70 (1.17, 11.66)**
Sexual abuse history before age 16	33 (32.4)	1.71 (0.71, 4.11)	–

Statistically significant odds ratios boldfaced in Table 1, p ≤ 0.05.

*Models adjusted for age, number of pregnancies ever carried to full term. Adjusted models only run for statistically significant associations in column three of Table 1.

**Black, non-Latina compared to all other groups.

The 29 women drawn from the cross-sectional study who participated in semi-structured interviews were similar on demographic characteristics to the rest of the cross-sectional study sample. Their average age was 36, 79% (N = 23) were Black, and 14% (N = 4) were White; 69% (N = 20) had a high school education. Fifteen of the 29 women (51.7%) who participated in the interviews reported having had a tubal ligation.

### Factors associated with sterilization history

In unadjusted logistic models run on the cross-sectional survey data, age (OR = 1.12, 95% CI 1.06, 1.18), number of pregnancies ever carried to full term (OR = 2.10, 95% CI 1.50, 2.96), history of exchanging sex for money, drugs, or life necessities (OR = 2.77, 95% CI 1.08, 7.10), number of months incarcerated in lifetime (OR = 1.02, 95% CI 1.00, 1.04), past year drug dependence (OR = 2.33, CI 0.99, 5.52), history of alcohol problems (OR = 2.95, 95% CI 1.19, 7.33), and child physical abuse history (OR = 2.98, CI 1.22, 7.26) were associated with ever having reported sterilization (p ≤ 0.05, see Table 1). In models that adjusted for age and number of pregnancies ever carried to full term, the only factor associated with sterilization history was child physical abuse history (OR = 3.70, 95% CI 1.17, 11.66) (p ≤ 0.05, see Table 1).

In the sample of women who participated in semi-structured interviews, women who reported sterilization histories had spent on average of 11 months in jail or prison, compared to an average of one month for women who did not report a sterilization history. Women who reported sterilization histories in this sample had an average of three children (range 1–7), compared to one child for those who did not report a sterilization history. Thirty-one percent (N = 9) of women who were sterilized reported having a high school education/GED or some college. Women were on average 25 years old when they had the tubal ligation.

Four themes related to sterilization history were identified among the sample of participants who completed semi-structured interviews and reported sterilization histories: desire to limit childbearing, general support, financial concerns, and family history.

### Desire to limit childbearing

Most women who reported having had a tubal ligation said they simply didn’t want any more children. Marta^a^, a 33-year old, said, “My first two were ten months and two days apart. I just got pregnant really easy”. Another participant, June, a 35-year old, said, “I had two kids. Two kids! I didn’t want to just keep on having kids”. Shelby, a 42-year old, said, “I have six kids. I’ve had eight pregnancies. And three of ‘em were born out of my drug addiction. I wasn’t planning on having this last child, so I thought it was time to get my tubes tied”. Two participants indicated that they had gotten pregnant while using other forms of birth control, like condoms and birth control pills. Karen, a 43-year old, said that birth control pills made her gain weight, and the injection made her gain even more weight. As soon as she stopped her injections (because of weight gain), she became pregnant. After that she decided to get the tubal ligation. Bobbi, a 47-year old, said that when she had her tubal ligation at age 26 after having had one child, she hadn’t even considered taking other forms of birth control, such as pills. She said, “I didn’t want none of that. I just wanted em’ clamped”.

### General support

In general, participants reported that providers, partners, and their mothers were supportive of their decision to get a tubal ligation. Both Marta (above) and Corrie, a 42-year old, said that their partners/fathers of their children were supportive of the decision. Vada, a 44-year old participant, said her doctor brought it up and talked to her about it; both her husband and mother were supportive.

Shelby, a 42-year old, said her medical providers were supportive. She requested the procedure from her OB who had not previously offered sterilization as an option. Some participants instead said that their providers discouraged the procedure. For example, June (above) said that her provider tried to talk her out of it, arguing that she might find someone in the future with whom to have a baby. Similarly, Bobbi (above) said that her provider tried to talk her out of a tubal ligation after having had one child; the discussion included her age (26 years) and that she might someday want to get married, which she perceived to be the provider’s way of discouraging her.

Only one participant reported pressure from providers for medical reasons. Susan, a 31-year old said about the tubal ligation: “The worst mistake I ever made. I was 25. I had a stroke. I had a real bad high blood pressure. With clots. So I had to get it done. The doctors made me”. She also reported being on three medications, including an anticoagulant, and being told that her life was in danger. She acknowledged that she felt other types of birth control did not work for her. Despite her past use of both pills and the Depo shot, she had six children.

Other participants reported explicit pressure from their mothers to get the procedure done. Karen (above) said recalling her experience, “I was 19. He was my fourth kid. So my mom was like, ‘Get them tubes tied!’ So I did it because my mom asked me to. If I could do it over again, I probably wouldn’t have ‘em tied right now. Cuz I did want one more kid”. Similarly, when we asked another participant about her decision to have her tubes tied, she said, “Cuz, listening to my mother. My mother said, ‘Look you got three kids. They don’t do much but slow you down. Times are hard. And I just listened to my mom”.

### Financial concerns

Financial concerns raised by participants were in two categories: raising children and paying for birth control. A familiar refrain with participants who reported a sterilization history, was “times are hard”. For example, Diane, a 46-year old, recalled her decision to get a tubal ligation after her seventh child said, “At times I get depressed about it, because I love kids. And I want to have more kids. You know, and I’m with somebody that . . . I wouldn’t mind having a kid with. Then other times, I think about it. I’m kind of glad I can’t because times is hard right now. You know? It’s just hard taking care of another person”. Similarly, when we asked Diane if there was anyone who helped her make the decision, she said, “Their dad. He kinda disagreed with it. For awhile, for a long time, he didn’t want me to get it done. But it just seemed like every time I turned around, arg, I was pregnant and times were getting harder and harder”.

Another participant responded to our question about why she didn’t consider other types of long-acting reversible contraception she said, “I didn’t want no mishaps. I’m a convicted drug felon. So I wasn’t sure if I was going to be able to get Medicaid. I didn’t know if I would be able to keep up with the shot”. However, Medicaid covered the cost of her tubal ligation. Finally, Sally (above) said “I always have some type of protection ready. Cuz you don’t want to be held down. Times is hard. . . I don’t see nothing coming easy”.

### Family history

Four out of 15 of our participants with sterilization histories reported family members who had tubal ligations: Marta, Sally, Cara, and Karen reported that their mothers had tubal ligations. Cara’s sister had one after she did.

## Discussion

Sterilization is the second leading method of contraception used by women in the U.S. However, this method is not evenly distributed along racial/ethnic lines or educational status. Between 1998 and 2008, 15% of Caucasian women, 22% of African-American and 20% of Latina women reported sterilization as their contraceptive method [14]. In the period 2006–2008 among women who reported using sterilization as their contraceptive method, 55% of those women had less than a high school education compared to 16% of those who had college degrees [14]. Among incarcerated women in this study and others, between 31-40% of women reported use of sterilization as a contraceptive method [8], suggesting that women in the U.S. criminal justice system report sterilization use at higher rates than the general population.

These trends forced us to consider the ways in which the vulnerable group of women with criminal justice histories chose sterilization and the extent to which those choices were constrained. Our qualitative analysis suggested that women opted for sterilization because of an explicit desire to limit childbearing and concern about the financial implications of not taking such action, findings reflected in the literature [32]. For many participants, the opinions and experiences of their mother were important in shaping women’s decisions, which has also been reported among non-incarcerated samples of women [33]. Only one participant in our study reported coercion from medical providers (albeit while ignoring her serious medical problems), though such abuses have been recently documented among incarcerated women [14]. The quantitative analysis found a statistically significant relationship between reproductive decision-making and childhood physical, but not sexual abuse. This latter finding is potentially new to the literature and may have important relevance for incarcerated women, many of whom have trauma histories [2]. That women with these histories of trauma and vulnerability may be more likely to have undergone the permanent procedure of sterilization raises questions about the extent to which they were more vulnerable to experiencing coercion around the procedure. Alternatively, it is unclear if negative childhood experiences have shaped these women’s desires about their own childrearing decisions.

As described above, we found little evidence that women in our sample had been explictly coerced into undergoing sterilization. However, more implicit constraints, such as their interpersonal relationship histories – including childhood abuse histories – were associated with sterilization use. The association between partner violence and contraceptive non-use has been previously documented, as well as the impact of childhood sexual abuse and women’s sexual risk behaviors [34, 35]. Familial pressure, from mothers in particular, also seemed to play an important role in the decision for sterilization. These individual level variables are complicated by dissatisfaction or inability to maintain use of the most traditional contraceptive methods of oral contraceptives, injections, and condoms among our sample, specifically, and incarcerated women in general [8]. We suggest that difficulties in accessing contraceptive services should be considered as a factor in erratic or inconsistent use of these methods and minimal use of more reliable but non-permanent contraception such as intrauterine devices. The possibility that access may also be challenged by women with a history of abuse, who may actively avoid ongoing reproductive health care, must also be considered. It should be noted that financial constraints, which we found to be part of the women’s narrative in describing their choice for sterilization, may be unique to vulnerable groups, such as women with criminal justice histories who often have limited earning potential and financial resources [4, 5].

Limitations of the present study were its cross-sectional design and our secondary data analysis, which prohibited us from truly understanding the pathways to sterilization in this sample. For example, we did not systematically collect data about when women received tubal ligations, and whether long-acting reversibe contraceptives were readily available at the time as an alternative. Additionally, semi-structured interviews about sterilization history were not designed to specifically complement survey data collection. Though our study design and sampling strategies had limitations in this regard, we were able to conduct one of the few exploratory studies of factors associated with incarcerated women’s sterilization use [9]. Finally, the inclusion of participants from more than one jail, region, or in other countries, would allow generalization of the findings to a broader population of women and help us understand variations in practice and policy.

## Conclusions

Our findings suggest that though explicit coercion may not be a factor in incarcerated women’s choice for sterilization, interpersonal relationship histories, negative experiences with contraceptives, and structural constraints, such as financial concerns and ongoing criminal justice involvement seem to be factors in opting for sterilization among the vulnerable group of women with criminal justice histories. Public health programs that connect women to reproductive health services should acknowledge these constraints on contraceptive decision-making. The findings of this study do reflect the structural constraints suggested over thirty years ago, that “even when voluntary, sterilization is often chosen in context of heavy structural constraints” [36]. Women with criminal justice histories may opt for sterilization in the face of long trauma histories, limited financial resources, and pressure from female family members to limit childbearing, not to mention the nature of their ongoing criminal justice involvement. Family planning programs should balance these heavy structural constraints against the options for birth control for these vulnerable women.

## Endnote

^a^Pseudonyms were used for all participants.

## References

[1] West H, Sabol W (2009). Prison Inmates at Midyear 2008. Statistical Tables.

[2] Lynch S, DeHart D, Belknap J, Green B (2012). Women’s Pathways to Jail: The Roles and Intersections of Serious Mental Illness and Trauma.

[3] Butler T, Richters J, Yap L, Papanastasiou C, Richards A, Schneider K, Grant L, Smith AMA, Donovan B (2010). Sexual Health and Behaviour of Queensland Prisoners: With Queensland and New South Wales Comparisons.

[4] Freudenberg N, Daniels J, Crum M, Perkins T, Richie BE (2008). Coming home from jail: the social and health consequences of community reentry for women, male adolescents, and their families and communities. Am J Public Health.

[5] Freudenberg N, Moseley J, Labriola M, Daniels J, Murrill C (2007). Comparison of health and social characteristics of people leaving New York City jails by age, gender, and race/ethnicity: implications for public health interventions. Public Health Rep.

[6] Harlow C (2003). Education and Correctional Populations.

[7] The Sentencing Project (2012). Incarcerated Women.

[8] Clarke JG, Hebert M, Rosengard C, Rose J, Da Silva K, Stein M (2006). Reproductive health care and family planning needs among incarcerated women. Am J Public Health.

[9] Pruitt SL, Von Sternberg K, Velasquez MM, Mullen PD (2010). Condom use among sterilized and nonsterilized women in county jail and residential treatment centers. Womens Health Issues.

[10] Reilly P (1991). The Surgical Solution: A History of Involuntary Sterilization in the United States.

[11] Davis A (1981). Women, Race, and Class.

[12] Gordon L (1977). Woman’s Body, Woman’s Right: A Social History of Birth Control in America.

[13] U.S. Department of Health & Human Services (1978). C.F.R. Section 441.250-259, Chapter IV.

[14] McGreevy P, Willon P (2013). Female inmate surgery broke law.

[15] Canadian Broadcasting Corporation (1999). Alberta apologizes for forced sterilization.

[16] New York Times (2003). For sterilization, target is women.

[17] Amnesty International (2010). Thousands at risk of forced sterilization in China.

[18] Mosher WD, Jones J (2010). Use of Contraception in the United States: 1982–2008. Vital and health statistics. Series 23, Data from the National Survey of Family Growth.

[19] Chambliss DF, Schutt RK (2010). Making Sense of the Social World: Methods of Investigation.

[20] Zhang W, O’Brien N, Forrest JI, Salters KA, Patterson TL, Montaner JS, Hogg RS, Lima VD (2012). Validating a shortened depression scale (10 item CES-D) among HIV-positive people in British Columbia, Canada. PLoS ONE.

[21] Compton W, Grant B, Colliver J, Glanz MD, Stinson FS (2004). Prevalence of marijuana disorders in in the United States: 1991–1992 and 2002–2002. JAMA.

[22] Ewing J (1984). Detecting alcoholism: The CAGE questionnaire. JAMA.

[23] Sherin KM, Sinacore JM, Li XQ, Zitter RE, Shakil A (1998). HITS: A short domestic violence screening tool for use in a family practice setting. Fam Med.

[24] Walsh C, MacMillan H, Trocme N, Jamieson E, Boyle M (2008). Measurement of victimization in adolescence: Development and validation of the Childhood Experiences of Violence Questionnaire. Child Abuse Negl.

[25] Borrero S, Moore C, Qin L, Schwarz E, Akers A, Creinin M, Ibrahim S (2009). Unintended pregnancy influences racial disparity in tubal sterilization rates. J Gen Intern Med.

[26] Morgan SP, Teachman JD (1988). Logistic regression: description, examples, and comparisons. J Marriage Fam.

[27] Katz J, Smelser N, Baltes P (2001). Analytic Induction. International Encyclopedia of the Social and Behavioral Sciences.

[28] Krippendorff K (1980). Content analysis: An introduction to its methodology.

[29] Miles MB, Huberman AM (1984). Qualitative data analysis: A sourcebook of new methods.

[30] Pascale C (2010). Cartographies of Knowledge: Exploring Qualitative Epistemologies.

[31] Clark JP, Godlee F, Jefferson T (2003). How to peer review a qualitative manuscript. Peer Review in Health Sciences.

[32] Borrero S, Nikolajski C, Rodriguez KL, Creinin MD, Arnold RM, Ibrahim SA (2009). “Everything I know I learned from my mother…Or not”: perspectives of African-American and white women on decisions about tubal sterilization. J Gen Intern Med.

[33] Borrero S, Abebe K, Dehlendorf C, Schwarz EB, Creinin MD, Nikolajski C, Ibrahim S (2011). Racial variation in tubal sterilization rates: role of patient-level factors. Fertil Steril.

[34] Arias I (2004). The legacy of child maltreatment: Long-term health consequences for women. J Womens Health.

[35] Williams C, Larsen U, McCloskey L (2008). Intimate partner violence and women’s contraceptive use. Viol Against Women.

[36] Petchesky R (1979). Reproduction, ethics, and public policy: The federal sterilization regulations. Hastings Cent Rep.

[CR37] The pre-publication history for this paper can be accessed here:http://www.biomedcentral.com/1471-2458/14/773/prepub

